# Retroviral Elements in Human Evolution and Neural Development

**Published:** 2021

**Authors:** Tongguang Wang, Tara T. Doucet-O’Hare, Lisa Henderson, Rachel P. M. Abrams, Avindra Nath

**Affiliations:** 1 Translational Neuroscience Center, National Institute of Neurological Disorders and Stroke, National Institutes of Health, Bethesda, Maryland, 20892, USA; 2 Section of Infections of the Nervous System, National Institute of Neurological Disorders and Stroke, National Institutes of Health, Bethesda, Maryland, 20892, USA

Human embryogenesis and the development of its most unique product, the human brain, are believed to be precisely regulated by factors adopted during human evolution that differentiate us from other species. Nevertheless, increasing evidence shows an unthinkable “alien” factor may have contributed to the process. Pervasive horizontal gene transfer between species mediated by retroviruses is such a defining factor of evolution [1]. Retroviral infections occurred in germline cells and were able to transfer the genomic codes vertically from parent to offspring. These genes once integrated into the host chromosome, can get dispersed and exist in multiple mutated copies throughout the host genome. As a result, retroviral genes and other retro elements contribute to about 50% of the human genome. Of these, 20% belong to the group of LINEs and over 8% consists of HERVs which are relatively intact since they were acquired more recently [2]. From an evolutionary point of view, these retroviral elements have at least a few known functions that could benefit the human host. Generally, the vast amount of such “relic” genes in the genome can provide a specific buffer zone to preserve functional genes against further viral infections and other gene mutation causing events. The similarities of gene sequences and functions provide a more specific competition to limit further similar viral infections [3]. These functions are evidenced by the abnormal shares of mutations and translocations within the retroviral elements compared with other functional genes. Other functions of the HERV proteins lent to the host include the immune regulatory functions, such as an immunosuppressive function mediated by a domain located in the transmembrane subunit of the HERV-W [4,5]. In the present review, we focus on the effects of retroviral elements on human embryogenesis and neural development.

## Retroviral Elements and Human Embryogenesis

Although mostly silenced in somatic cells, increased activities of retroviral and other transposable elements have been observed during embrogenesis and in pluripotent stem cells. These retro elements include LINE-1 [6–8], the older HERVs, and the most recently incorporated HERV, HERV-K, which is also the best-preserved family in the human genome [9–11]. The oldest full-length envelope gene identified to date in humans, HEMO [human endogenous MER34 (medium-reiteration-frequency-family-34) ORF], was captured as part of a retrovirus, MER34 which was incorporated into the mammalian genome more than 100 Mya [12]. This virus has a preserved open reading frame for only the envelope gene. This protein is detectable in the blood of pregnant women and is highly expressed in pluripotent stem cells and tumors [12]. Other relatively old HERV genes include two almost identical HERV-V envelope genes in chromosome 19, ENVV1 and ENVV2. These viral genes are found in simian species and humans but not in the pro-simian species. ENVV1 has a 477 amino-acid long open reading frame and ENVV2 has an open reading frame containing 535 amino acids. Variation is only observed in the C-terminus of the genes by a ~60 amino-acid truncation of *ENVV1*, due to a one nucleotide insertion leading to a frame shift. Both ENVV1 and ENVV2 show placenta-specific expression in humans and a baboon species [13]. HERV-R (ERV3) envelope also expresses in human placenta, as well as in developing tissues, such as the adrenal cortex, kidneys, tongue, heart, liver and CNS [14].

HERV transcripts are increased during cell transformation and in human pluripotent stem cells [15]. We compared the expression of HERV elements in induced pluripotent stem cells (iPSCs) and differentiated neural cells using RNA-Seq analysis [16]. We found that 4,305 HERV annotated regions of the Human Genome (hg38) were expressed in at least one cell type profiled; 1302 regions were expressed exclusively in iPSCs and 574 regions were differentially expressed between one or more cell types. Most of the differential expressions were between iPSCs and other cell types, suggesting that maximal expression of these genes occurs in early embryogenesis, then they get silenced during neural development.

The mechanisms of retro elements influencing embryonic development are just being realized. The LTRs function in a stage-specific manner by activating transcription, altering protein-coding sequences, producing noncoding RNAs, and even supporting the evolution of new protein-coding genes, resulting in mRNAs, lncRNAs, or proteins with regulatory roles (46). In the 2 cell-stage, many gene’ transcripts are initiated from ERV LTRs, such as MERVL (47). Some ERV families contain preserved splice sites that join the ERV segment with non-ERV exons in their genomic vicinity (48). The envelope protein of HERV-W is expressed in mammals as syncytin. It has been adopted by human species as an essential cellular protein for trophoblast and placental development [1,17,18]. Another HERV, HERV-Fb1, transcribes the protein suppressyn, which only expresses in the placenta *in vivo*, competes with sycytin-1 for its receptor (ASCT-2), and inhibits sycytin-1 mediated trophoblast cell-cell fusion [19]. HERV-H activation is another marker for pluripotent stem cells [20] and plays a regulatory role in the stemness and differentiation potential of pluripotent stem cells [21]. HERV-H is expressed as a non-coding RNA that regulates transcriptional factors [22]. Along these lines, we have shown that HERV-K subtype HML-2 envelope protein is expressed in human pluripotent stem cells but not in differentiated neural cells. HERV-K envelope regulates stemness and the differentiation potential of the cells by interacting with cell membrane molecules and cell matrix networks, leading to the activation of signaling mechanisms implicated in cell proliferation [16].

## Retroviral Elements and Neural Development

Recently acquired HERV-K subfamily HML-2 has nearly full-length viral sequences with open reading frames for *gag*, *pro*, *pol*, and *env* genes [23]. HML-2 activation has been observed in pluripotent stem cells [24], mesenchymal stem cells [25] and certain tumors [26]. We found that HML-2 envelope expression increased in pluripotent stem cells but diminished during neuronal differentiation [16]. When HML-2 envelope was forced to express by transfecting iPSCs with an HML-2 *env* containing plasmid, the neuronal induction process was inhibited, as indicated by the cells’ morphology and lower nestin expression. Nestin is a marker of neural stem cells but is not expressed in pluripotent stem cells. On the contrary, inhibition of HML-2 *env* in iPSCs facilitated induction of neural stem cells and eventually neuronal differentiation [16].

LINE-1s are abundant retrotransposons that comprise approximately 20% of mammalian genomes. Activation of LINE-1 occurs mainly in early embryonic development and during hippocampal neurogenesis [27]. Active LINE-1 retrotransposons can create insertions [28], deletions, and new splice sites to the genome [27,29]. Somatic LINE-1 retrotransposition during neurogenesis is a source of genotypic variation among neurons. The highest concentration of such LINE-1 activation in the human adult brain is in the dentate gyrus, the hotspot of adult neurogenesis [30]. A single-cell retrotransposon capture sequencing (RC-seq) study on individual human hippocampal neurons estimated that 13.7 somatic LINE-1 insertions occurred per hippocampal neuron. These genomic loci carried the sequence hallmarks of target-primed reverse transcription, suggesting pervasive LINE-1 mosaicism in hippocampal neurons [31].

## Mechanisms of HERV Control During Embryonic and Neural Development

There are many mechanisms that regulate HERV expression throughout development and in differentiated cells. Transcriptional activity of HERVs is regulated by binding of epigenetic modifiers and transcription factors to the long terminal repeats (LTRs) that flank the 5’ and 3’ ends of the viral genome. Many HERVs exist as only solo LTRs, most likely due to homologous recombination between the 5’ and 3’ LTRs that results in deletion of the internal coding sequence [32]. Transcription profiles of HERV expression in healthy human tissues indicate that HERV proviruses that retain intact coding sequences are differentially expressed in a cell type specific manner, with the highest expression observed in the thyroid glands, skin, reproductive organs and tissues of embryonic origin, and the lowest expression in non-dividing, terminally differentiated cells [33].

Aberrant expression of endogenous retroviral genes at an inappropriate embryonic stage or in tissues where their transcription is normally suppressed is strongly associated with development of disease. Therefore, control of HERV expression by epigenetic regulation is a crucial part of tissue homeostasis. DNA (CpG) methylation performed by DNA methyltransferase 1 (DNMT1) is an important mechanism of retroelement silencing, particularly for the most intact and transcriptionally active member, HERV-K/HML-2; the LTRs of HERVs are often hypermethylated in healthy tissues, and loss of CpG methylation results in increased HML-2 transcription that may contribute to oncogenesis [34,35]. In addition to DNA methylation, chromatin remodeling due to acetylation or methylation of histone tails plays an important role in regulating the accessibility of the LTR to transcription factors and other proteins. Specifically, trimethylation of lysine 9 on histone H3 (H3K9Me3) by the lysine methyltransferase SETDB1 is strongly associated with repression of both HERV and LINE-1 elements. Loss of this histone mark resulted in global upregulation of retroelements in colorectal cancer cells [36]. These methylation patterns are established during embryogenesis when members of the Krüppel associated box zinc finger protein (KRAB-ZFP) family, such as TRIM28/KAP1, bind to HERV promoters in a sequence-specific manner. A repressive complex that includes DNMTs and SETDB1 is then recruited to induce formation of heterochromatin [37].

It has been hypothesized that nucleosomal positioning plays a role in HERV transcription by a mechanism observed in latent HIV infections. In a transcriptionally inactive HIV promoter, there is a nucleosome positioned immediately downstream of the transcription start site, which prevents access to transcriptional machinery. Binding of specific transcription factors to enhancers in the LTR or differential methylation or acetylation of the histones within the nucleosome induces repositioning of the nucleosome to allow for transcription [38]. HERV-K/HML-2, contains many intact promoter and enhancer elements in its LTRs that can regulate its expression [39]. Importantly, there is genetic variation in the LTR among HML-2 elements in the genome; therefore, the promoter, enhancer, and transcription factor binding sites at a given locus are variable. There is a positive correlation between HERV-K/HML-2 LTR sequence variation and promoter expression patterns [40]. Sequencing data from the 1000 Genomes Project, observed that an active form of the chromosome 11p15.4 HML-2 locus was polymorphic in the human population with an allele frequency of 51% [40]. In addition, locus, 3q12.3 was observed to be fixed in humans but absent from the orthologous virus in chimpanzees and gorillas [40]. These data suggest that transcription factor binding site differences between HML-2 LTRs may play a role in the differential expression seen among individuals.

Recent work in breast cancer cell lines has revealed a role for the progesterone-response element and the octamer-binding transcription factor 4 binding sites in some HML-2 LTRs [41]. An isoform of the progesterone receptor was found to bind the progesterone-response element in the LTR of HML-2. This binding was mediated by a physical interaction between the progesterone receptor and octamer-binding transcription factor 4 transcription factor [41]; however, the role of this interaction in brain development or disease has not yet been studied. The HML-2 promoter also contains several putative binding sites for the ubiquitously expressed RNA-binding protein TDP-43, which is dysregulated in neurodegenerative diseases such as ALS and FTLD [42]. Overexpression of TDP-43 in iPSC-derived neurons caused upregulation of HML-2 transcripts and induced cytotoxicity, which suggests that TDP-43 may function as a transcriptional regulator of HERVs in the central nervous system. The HERV-K LTR, also contains multiple transcriptional initiator sites, which can be targeted by other transcription factors such as microphthalmia-associated transcription factor. The latter is responsible for the significantly enhanced HERV-K expression in malignant melanoma [43]. In another case, an HML-2 insertion upstream of *PRODH* gene exhibits tissue-specific enhancer activity with maximal expression in hippocampus. The enhancer activity is regulated by methylation and involves the binding of SOX2. PRODH is the mitochondrial proline dehydrogenase that regulates proline catabolism. PRODH is critical for normal CNS function and has been associated with schizophrenia [44].

## Role of Envelope Proteins in Development

Retroviral envelope proteins are known to facilitate cell-to-cell adhesion and in some cases cause cell fusion. For example, HERV-W syncytin leads to fusion of trophoblasts to form the placenta [17,45]. Interestingly, HERV proteins often use membrane transport proteins as receptors, such as Alanine, Serine, Cysteine Transporter 2 (also named SLC1A5) for syncytin-1 (HERV-W) [46, 47], and CD98 heavy chain, also named solute carrier family 3 member 2 for HERV-K envelope [16]. Interactions between HML-2 envelope and the CD98 heavy chain leads to activation of signaling pathways in pluripotent stem cells to maintain cell adhesion and stem cell morphology [16]. Amongst them are the mTOR and LPCAT1 pathways. LPCAT1 is downstream of mTOR and catalyzes the conversion of lysophosphatidlycholine to phosphatidylcholine [48] and the palmitolysation of histone H4 to open the chromatin and maintain stemness [49]. HML-2 envelope expression is also associated with expression of ribosomal protein S6 (rpS6), a crucial effector of the mTOR signaling pathway [16]. Active mTOR regulates cell size [50] and cell proliferation by increasing protein synthesis and regulating ribosome biogenesis and autophagy, subsequently affecting the cytosol viscosity [51]. Thus HML-2 envelope is also critical for pluripotent stem cell function by regulating cell functions through the evolutionarily conserved mTOR pathway. Increased levels of mTOR activation in human outer subventricular zone radial glia is a critical factor in the differentiation of human brains from non-human primates [52]. We observed that transfection of rhesus neural stem cells with HML-2 *env* resulted in high levels of rpS6 and LPCAT1 expression, further implying that HML-2 incorporation in the human genome caused the increased activity of mTOR in human stem cells and may have played a role in human brain evolution [16].

## Role of Gag Proteins in Synaptic Function

The neuronal gene Arc/Arg3.1 plays an essential role in the consolidation of synaptic plasticity and long-term memory [53] and the dysregulation of Arc is implicated in cognitive diseases [54]. Arc is highly conserved among mammals, birds, reptiles, and amphibians, but is not present in fish [55,56]. A crystal structure of the Arc protein revealed significant homology to the capsid region of the human immunodeficiency virus (HIV) gag protein [55]. Furthermore, Arc is known to contain an internal ribosomal entry site, a feature common in the translation of viral genes [57]. Sequence analysis indicated that Arc evolved from the Ty3/gypsy family of retrotransposons, which are present in animal, plant, and fungal kingdoms [58]. During the domestication of Arc in vertebrates, the N-terminal domain, which mediates binding with synaptic proteins was acquired, and the zinc knuckle and reverse transcriptase portions were lost [55,58].

In the presence of RNA, purified Arc protein spontaneously assembles into oligomeric, virus-like capsid structures [56]. Arc protein interacts with and binds to its own mRNA. Through the release of synaptic vesicles, Arc transfers its mRNA intercellularly between neurons. This mRNA can then be translated locally in the dendrites [56]. Disruption of this transfer in Drosophila results in aberrations in synapse maturation and activity-dependent plasticity [59]. Tetrapod and fly Arc genes originated independently, from distinct lineages of Ty3/gypsy retrotransposons, but both are involved in intercellular trafficking of RNA and are essential for proper neuronal function [56,59]. The human genome contains at least 85 genes that encode for proteins resembling viral gag proteins [58]. Therefore, it is likely that there are additional retroviral gag elements that have been coopted for essential physiological functions. Additionally, it is possible that other viral elements, such as the viral protease, may have played a role in the evolution of the human nervous system.

## Mechanism of LINE-1 Regulation in Early Human Embryogenesis and Neural Development

The RNA of LINE-1 is localized to the nucleus in embryonic stem cells and pre-implantation embryos. DMNT-1 is responsible for the high CpG methylation of LINE-1 lineages that are younger than 12.5 million years, corresponding to hominoid-specific elements, including many that are human-specific. Deletion of DMNT-1 resulted in hypomethylation of DNA and chromatin remodeling and increased activation of these younger LINE-1s, which are responsible for transcriptional enhancement of many protein-coding genes involved in neuronal functions and psychiatric disorders [60]. LINE-1 neuronal transcription and retrotransposition are also increased in the absence of methyl-CpG-binding protein 2, another protein involved in global DNA methylation [61].

LINE-1 functions as a nuclear RNA scaffold, which recruits Nucleolin and Kap1/Trim28 to repress Dux, the master activator of a transcriptional program specific to the 2-cell embryo. In humans, there is a strong association between the establishment of accessible chromatin and embryonic genome activation. A large proportion of the early activated genes and HERVs are bound by DUX4 and become accessible as early as the 2- to 4-cell stages [62].

Many transcription factors important to neural development regulate, or are regulated by, LINE-1. For example, one LINE-1 element contains overlapping Sox2 and T-cell factor/lymphoid enhancer factor (TCF/LEF)-binding sites (Sox/LEF), which make up a transcriptional site regulated by Wnt and β-catenin signals. Wnt3a ligand and β-catenin increased acetylated histone H3 levels in the LINE-1 genomic region, inducing the active chromatin state, causing an increase in the amount of LINE-1 ORF2 mRNA in neuronal stem cells. Wnt3a ligand and β-catenin signaling may also affect nearby genes such as DCX and neurogulin-4, which are important in regulating neural development [63]. The SOX-11 protein also binds the LINE-1 promoter, causing induction of LINE-1 transcription in neural differentiating conditions [64].

## Summary and Future Directions

We have presented evidence from recent literature that retroviral elements incorporated in the human genome have played important roles during human evolution. Although most of the insertions have been silenced, a few of the retroviral elements such as HERVs and LINE-1s can still be active in human embryogenesis and play important functions in human development. The fine regulation of HERV-K and LINE-1 are especially important in human brain development. HERV-K activation is a key event that regulates mTOR activation, which differentiates human beings from other primates. Additionally, the increased LINE-1 activity during neural genesis mediates adult neuron diversity in the brain.

The regulation of the retroviral elements is influenced by a general mechanism regulating DNA methylation status, which is lower in certain stages of embryogenesis. There are also regulatory mechanisms specific to species, tissues, even cell types leading to activation of individual retroviral elements. It is notable that there are also interactions among retroviral elements, which may be either positive or negative associations. Our knowledge of the role of retroviral elements in brain development and disease is still rudimentary but this is a fertile and promising area of investigation that can provide novel insights into disease pathogenesis and identify new targets for treatment.

## Figures and Tables

**Figure 1:**
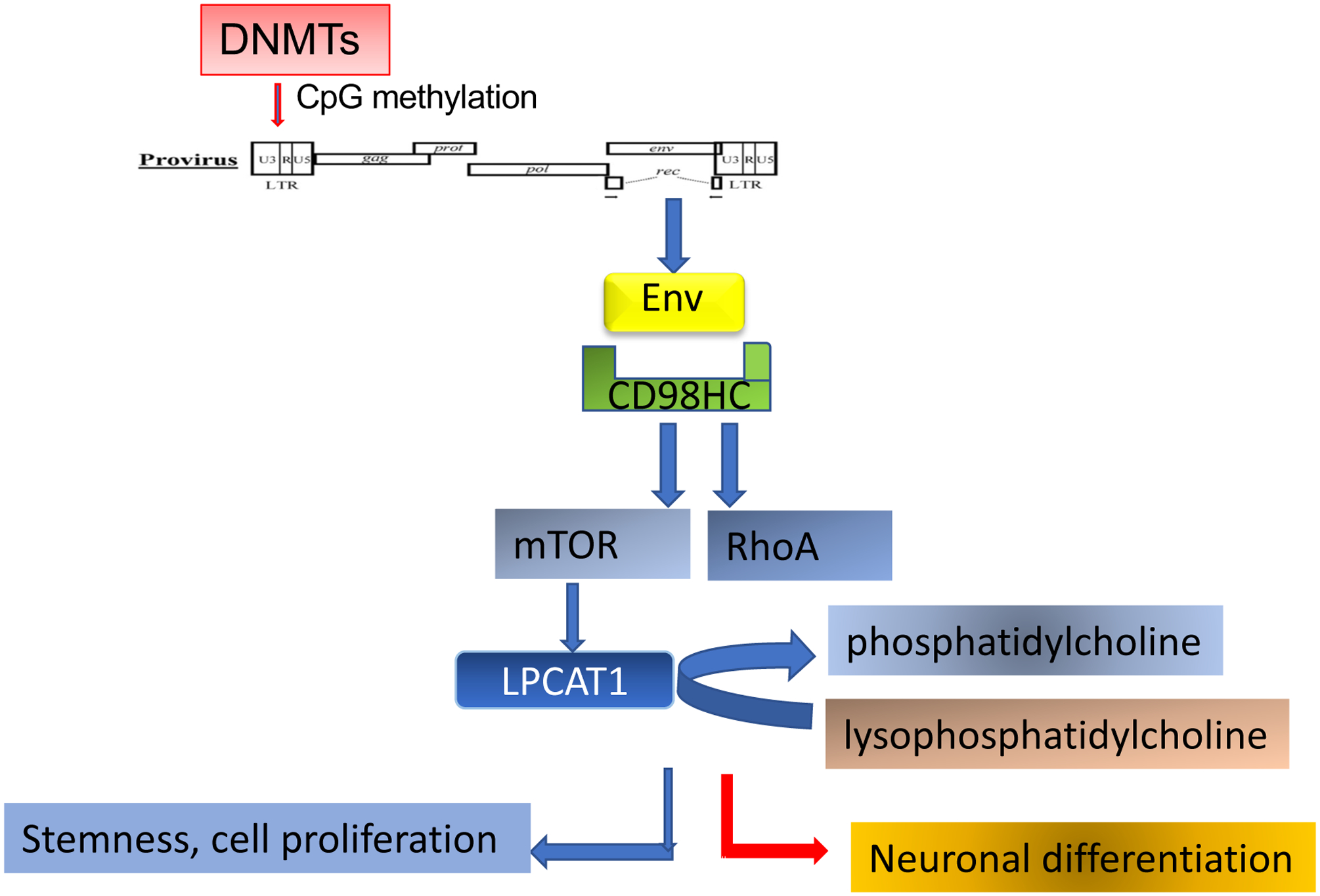
HERV-K Env regulation of neuronal differentiation. DNMTs mediates CpG methylation of HERV-K LTR, which leads to inhibition of the expression of the envelope. The decreased interaction of Env with CD98HC results in the inactivation of mTOR and LPCAT1 pathways, leading to decreased cell proliferation and the loss of stemness. Instead, the inhibition of RhoA and increase of LPC/PC ratio would facilitate neuronal differentiation. The blue arrow indicates decreased activities, and the red arrows indicate increased activities.

**Table 1:** Retro elements activation and function in human embryogenesis and nervous system development.

Retro elements	Expression	Functions
Human endogenous MER34 ORF (HEMO)	blood of pregnant women, pluripotent stem cells and tumors [12]	stemness marker
HERV-V env genes (ENVV1 and ENVV2)	placenta-specific expression in human and a baboon species [13]	possible beneficial role to the host
HERV-R (ERV3) env	human placenta, developing tissues such as adrenal cortex, kidneys, tongue, heart, liver and CNS [14]	non-essential role in regulating cellular differentiation such as in trophoblast differentiation
HERV-W Env protein syncytin	trophoblast and placenta [1,17,18]	fusion of the cytotrophoblasts to form the syncytia layer of placenta; immunosuppression
HERV-Fb1 transcribed protein suppressyn	placenta	competes with sycytin-1 for receptor ASCT-2, and inhibits sycytin-1 mediated trophoblast cell-cell fusion [19]
HERV-H (noncoding RNA)	placenta, pluripotent stem cells [20]	marker for stemness, regulates pluripotent stem cell related transcriptional factors [22]
HERV-K (HML-2 Env protein)	placenta, germ cells and pluripotent stem cells [16], mesenchymal stem cells [25] and certain tumors [26]	binds with CD98HC, activation of mTOR and LPCAT1 to regulate stemness and neuronal differentiation [16]
LINE-1	early embryogenesis, adult hippocampal neurons [27]	introduces insertions [28], deletions, new splice sites into the genome [27,29]; as a nuclear RNA scaffold which recruits Nucleolin and Kap1/Trim28 to repress Dux; mosaicism in hippocampal neurons

## Abbreviations:

ALS: Amyotrophic Lateral Sclerosis
Arc/Arg3.1: Activity-regulated cytoskeleton-associated protein
ASCT-2: Alanine-serine-cysteine Transporter 2
CD98HC: CD98 Heavy Chain
CNS: Central Nervous System
CpG: 5’-C-phosphate-G-3’
DCX: Doublecortin
DNMT: DNA Methyltransferase
Dux: Double homeobox
Env: Envelope
ENVV1: Endogenous retrovirus group V member 1, envelope
ENVV2: Endogenous retrovirus group V member 2, envelope
ESC: Embryonic Stem Cell
FTLD: Frontotemporal Lobar Degeneration
H3K9Me3: Trimethylation of lysine 9 on histone H3
HEMO: Human Endogenous MER34 (medium-reiteration-frequency-family-34) ORF
HERV: Human Endogenous Retrovirus
HIV: Human Immunodeficiency Virus
IncRNAs: Long none-coding RNAs
iPSC: Induced Pluripotent Stem Cell
KRAB-ZFP: Krüppel Associated Box Zinc Finger Protein
LPCAT1: Lysophosphatidylcholine Acyltransferase 1
LINE: Long Interspersed Nuclear Element
LTR: Long Terminal Repeat
MeCP2: Methyl-CpG-binding Protein 2
MERVL: Murine Endogenous Retroviral L
MITF: Microphthalmia-associated Transcription Factor
mTOR: Mammalian Target of Rapamycin
Mya: Million Years Ago
OCT4: Octamer-binding Transcription factor 4
ORF: Open Reading Frame
PRE: Progesterone-response Element
PRODH: Proline Dehydrogenase, mitochondrial
RC-Seq: Retrotransposon Capture Sequencing
rpS6: Ribosomal protein S6
SCL3A2: Solute Carrier family 3 member 2
SETDB1: SET domain Bifurcated histone lysine methyltransferase 1
Sox2: SRY (sex determining region Y)-box 2
TCF/LEF: T-cell Factor/Lymphoid Enhancer Factor
TDP43: TAR DNA-binding Protein 43
TRIM28/KAP1: Tripartite Motif Containing 28/ KRAB-associated Protein-1

## References

[1] Koonin EV, Wolf YI. Evolution of microbes and viruses: a paradigm shift in evolutionary biology?. Frontiers in Cellular and Infection Microbiology. 2012 Sep 13;2:119. 22993722 10.3389/fcimb.2012.00119 PMC3440604

[2] International Human Genome Sequencing Consortium. Initial sequencing and analysis of the human genome. Nature. 2001;409:860–921. 11237011 10.1038/35057062

[3] Ponferrada VG, Mauck BS, Wooley DP. The envelope glycoprotein of human endogenous retrovirus HERV-W induces cellular resistance to spleen necrosis virus. Archives of Virology. 2003 Mar 1;148(4):659–75. 12664292 10.1007/s00705-002-0960-x

[4] Mangeney M, Renard M, Schlecht-Louf G, Bouallaga I, Heidmann O, Letzelter C, Placental syncytins: Genetic disjunction between the fusogenic and immunosuppressive activity of retroviral envelope proteins. Proceedings of the National Academy of Sciences. 2007 Dec 18;104(51):20534–9. 10.1073/pnas.0707873105 PMC2154466 18077339

[5] Mangeney M, de Parseval N, Thomas G, Heidmann T. The full-length envelope of an HERV-H human endogenous retrovirus has immunosuppressive properties. Journal of General Virology. 2001 Oct 1;82(10):2515–8. 11562544 10.1099/0022-1317-82-10-2515

[6] Hohn O, Hanke K, Bannert N. HERV-K (HML-2), the best preserved family of HERVs: endogenization, expression, and implications in health and disease. Frontiers in Oncology. 2013 Sep 20;3:246. 24066280 10.3389/fonc.2013.00246 PMC3778440

[7] Barbulescu M, Turner G, Seaman MI, Deinard AS, Kidd KK, Lenz J. Many human endogenous retrovirus K (HERV-K) proviruses are unique to humans. Current Biology. 1999 Aug 26;9(16):861–S1. 10469592 10.1016/s0960-9822(99)80390-x

[8] Medstrand P, Mager DL. Human-specific integrations of the HERV-K endogenous retrovirus family. Journal of Virology. 1998 Dec 1;72(12):9782–7. 9811713 10.1128/jvi.72.12.9782-9787.1998 PMC110489

[9] Klawitter S, Fuchs NV, Upton KR, Munoz-Lopez M, Shukla R, Wang J, Reprogramming triggers endogenous L1 and Alu retrotransposition in human induced pluripotent stem cells. Nature Communications. 2016 Jan 8;7:10286. 10.1038/ncomms10286 PMC4729875 26743714

[10] van den Hurk JA, Meij IC, del Carmen Seleme M, Kano H, Nikopoulos K, Hoefsloot LH, L1 retrotransposition can occur early in human embryonic development. Human Molecular Genetics. 2007 Jul 1;16(13):1587–92. 17483097 10.1093/hmg/ddm108

[11] Pi W, Yang Z, Wang J, Ruan L, Yu X, Ling J, The LTR enhancer of ERV-9 human endogenous retrovirus is active in oocytes and progenitor cells in transgenic zebrafish and humans. Proceedings of the National Academy of Sciences. 2004 Jan 20;101(3):805–10. 10.1073/pnas.0307698100 PMC321762 14718667

[12] Heidmann O, Béguin A, Paternina J, Berthier R, Deloger M, Bawa O, HEMO, an ancestral endogenous retroviral envelope protein shed in the blood of pregnant women and expressed in pluripotent stem cells and tumors. Proceedings of the National Academy of Sciences. 2017 Aug 8;114(32):E6642–51. 10.1073/pnas.1702204114 PMC5559007 28739914

[13] Kjeldbjerg AL, Villesen P, Aagaard L, Pedersen FS. Gene conversion and purifying selection of a placenta-specific ERV-V envelope gene during simian evolution. BMC Evolutionary Biology. 2008 Dec;8(1):1–1. 18826608 10.1186/1471-2148-8-266 PMC2567338

[14] Andersson AC, Venables PJ, Tönjes RR, Scherer J, Eriksson L, Larsson E. Developmental expression of HERV-R (ERV3) and HERV-K in human tissue. Virology. 2002 Jun 5;297(2):220–5. 12083821 10.1006/viro.2002.1428

[15] Subramanian RP, Wildschutte JH, Russo C, Coffin JM. Identification, characterization, and comparative genomic distribution of the HERV-K (HML-2) group of human endogenous retroviruses. Retrovirology. 2011 Dec 1;8(1):90. 22067224 10.1186/1742-4690-8-90 PMC3228705

[16] Wang T, Medynets M, Johnson KR, Doucet-O’Hare TT, DiSanza B, Li W, Regulation of stem cell function and neuronal differentiation by HERV-K via mTOR pathway. Proceedings of the National Academy of Sciences. 2020 Jul 28;117(30):17842–53. 10.1073/pnas.2002427117 PMC7395438 32669437

[17] Mi S, Lee X, Li XP, Veldman GM, Finnerty H, Racie L, Syncytin is a captive retroviral envelope protein involved in human placental morphogenesis. Nature. 2000 Feb;403(6771):785–9. 10693809 10.1038/35001608

[18] Dupressoir A, Lavialle C, Heidmann T. From ancestral infectious retroviruses to bona fide cellular genes: role of the captured syncytins in placentation. Placenta. 2012 Sep 1;33(9):663–71. 22695103 10.1016/j.placenta.2012.05.005

[19] Sugimoto J, Sugimoto M, Bernstein H, Jinno Y, Schust D. A novel human endogenous retroviral protein inhibits cell-cell fusion. Scientific reports. 2013 Mar 15;3:1462. 23492904 10.1038/srep01462 PMC3598002

[20] Santoni FA, Guerra J, Luban J. HERV-H RNA is abundant in human embryonic stem cells and a precise marker for pluripotency. Retrovirology. 2012 Dec 1;9(1):111. 23253934 10.1186/1742-4690-9-111 PMC3558390

[21] Ohnuki M, Tanabe K, Sutou K, Teramoto I, Sawamura Y, Narita M, Dynamic regulation of human endogenous retroviruses mediates factor-induced reprogramming and differentiation potential. Proceedings of the National Academy of Sciences. 2014 Aug 26;111(34):12426–31. 10.1073/pnas.1413299111 PMC4151758 25097266

[22] Wildschutte JH, Williams ZH, Montesion M, Subramanian RP, Kidd JM, Coffin JM. Discovery of unfixed endogenous retrovirus insertions in diverse human populations. Proceedings of the National Academy of Sciences. 2016 Apr 19;113(16):E2326–34. 10.1073/pnas.1602336113 PMC4843416 27001843

[23] Fuchs NV, Loewer S, Daley GQ, Izsvák Z, Löwer J, Löwer R. Human endogenous retrovirus K (HML-2) RNA and protein expression is a marker for human embryonic and induced pluripotent stem cells. Retrovirology. 2013 Dec 1;10(1):115. 24156636 10.1186/1742-4690-10-115 PMC3819666

[24] Mareschi K, Montanari P, Rassu M, Galliano I, Daprà V, Adamini A, Human Endogenous Retrovirus-H and K Expression in Human Mesenchymal Stem Cells as Potential Markers of Stemness. Intervirology. 2019;62(1):9–14. 31104062 10.1159/000499185

[25] Schmitt K, Reichrath J, Roesch A, Meese E, Mayer J. Transcriptional profiling of human endogenous retrovirus group HERV-K (HML-2) loci in melanoma. Genome Biology and Evolution. 2013 Feb 1;5(2):307–28. 23338945 10.1093/gbe/evt010 PMC3590776

[26] Thomas CA, Paquola AC, Muotri AR. LINE-1 retrotransposition in the nervous system. Annual Review of Cell and Developmental Biology. 2012 Nov 10;28:555–73. 10.1146/annurev-cellbio-101011-155822 23057747

[27] Takeda K, Hozumi H, Nakai K, Yoshizawa M, Satoh H, Yamamoto H, Insertion of long interspersed element-1 in the M itf gene is associated with altered neurobehavior of the black-eyed white M itfmi-bw mouse. Genes to Cells. 2014 Feb;19(2):126–40. 24304702 10.1111/gtc.12117

[28] Richardson SR, Morell S, Faulkner GJ. L1 retrotransposons and somatic mosaicism in the brain. Annual Review of Genetics. 2014 Nov 23;48:1–27. 10.1146/annurev-genet-120213-092412 25036377

[29] Kurnosov AA, Ustyugova SV, Nazarov VI, Minervina AA, Komkov AY, Shugay M, The evidence for increased L1 activity in the site of human adult brain neurogenesis. PLoS One. 2015 Feb 17;10(2):e0117854. 25689626 10.1371/journal.pone.0117854 PMC4331437

[30] Upton KR, Gerhardt DJ, Jesuadian JS, Richardson SR, Sánchez-Luque FJ, Bodea GO, Ubiquitous L1 mosaicism in hippocampal neurons. Cell. 2015 Apr 9;161(2):228–39. 25860606 10.1016/j.cell.2015.03.026 PMC4398972

[31] Johnson WE. Origins and evolutionary consequences of ancient endogenous retroviruses. Nature Reviews Microbiology. 2019 Jun;17(6):355–70. 30962577 10.1038/s41579-019-0189-2

[32] Seifarth W, Frank O, Zeilfelder U, Spiess B, Greenwood AD, Hehlmann R, Comprehensive analysis of human endogenous retrovirus transcriptional activity in human tissues with a retrovirus-specific microarray. Journal of Virology. 2005 Jan 1;79(1):341–52. 15596828 10.1128/JVI.79.1.341-352.2005 PMC538696

[33] Szpakowski S, Sun X, Lage JM, Dyer A, Rubinstein J, Kowalski D, Loss of epigenetic silencing in tumors preferentially affects primate-specific retroelements. Gene. 2009 Dec 15;448(2):151–67. 19699787 10.1016/j.gene.2009.08.006 PMC2783545

[34] Lavie L, Kitova M, Maldener E, Meese E, Mayer J. CpG methylation directly regulates transcriptional activity of the human endogenous retrovirus family HERV-K (HML-2). Journal of Virology. 2005 Jan 15;79(2):876–83. 15613316 10.1128/JVI.79.2.876-883.2005 PMC538560

[35] Rajagopalan D, Tirado-Magallanes R, Bhatia SS, Teo WS, Sian S, Hora S, TIP60 represses activation of endogenous retroviral elements. Nucleic Acids Research. 2018 Oct 12;46(18):9456–70. 30053221 10.1093/nar/gky659 PMC6182167

[36] Hurst TP, Magiorkinis G. Epigenetic control of human endogenous retrovirus expression: focus on regulation of long-terminal repeats (LTRs). Viruses. 2017 Jun;9(6):130. 10.3390/v9060130 PMC5490807 28561791

[37] Peterson CL, Workman JL. Promoter targeting and chromatin remodeling by the SWI/SNF complex. Current Opinion in Genetics & Development. 2000 Apr 1;10(2):187–92. 10753786 10.1016/s0959-437x(00)00068-x

[38] Kovalskaya E, Buzdin A, Gogvadze E, Vinogradova T, Sverdlov E. Functional human endogenous retroviral LTR transcription start sites are located between the R and U5 regions. Virology. 2006 Mar 15;346(2):373–8. 16337666 10.1016/j.virol.2005.11.007

[39] Montesion M, Williams ZH, Subramanian RP, Kuperwasser C, Coffin JM. Promoter expression of HERV-K (HML-2) provirus-derived sequences is related to LTR sequence variation and polymorphic transcription factor binding sites. Retrovirology. 2018 Dec 1;15(1):57. 30126415 10.1186/s12977-018-0441-2 PMC6102855

[40] Durnaoglu S, Kim HS, Ahnn J, Lee SK. Human Endogenous Retrovirus K (HERV-K) can drive gene expression as a promoter in Caenorhabditis elegans. BMB Reports. 2020 Oct 31;53(10):521. 32867919 10.5483/BMBRep.2020.53.10.150 PMC7607151

[41] Holloway JR, Williams ZH, Freeman MM, Bulow U, Coffin JM. Gorillas have been infected with the HERV-K (HML-2) endogenous retrovirus much more recently than humans and chimpanzees. Proceedings of the National Academy of Sciences. 2019 Jan 22;116(4):1337–46. 10.1073/pnas.1814203116 PMC6347686 30610173

[42] Nguyen TD, Davis J, Eugenio RA, Liu Y. Female sex hormones activate human endogenous retrovirus type K through the OCT4 transcription factor in T47D breast cancer cells. AIDS Research and Human Retroviruses. 2019 Mar 1;35(3):348–56. 30565469 10.1089/AID.2018.0173

[43] Li W, Lee MH, Henderson L, Tyagi R, Bachani M, Steiner J, Human endogenous retrovirus-K contributes to motor neuron disease. Science Translational Medicine. 2015 Sep 30;7(307):307ra153–. 10.1126/scitranslmed.aac8201 PMC6344353 26424568

[44] Katoh I, Mírová A, Kurata SI, Murakami Y, Horikawa K, Nakakuki N, Activation of the long terminal repeat of human endogenous retrovirus K by melanoma-specific transcription factor MITF-M. Neoplasia. 2011 Nov 1;13(11):1081–IN42. 22131883 10.1593/neo.11794 PMC3223611

[45] Blaise S, de Parseval N, Bénit L, Heidmann T. Genomewide screening for fusogenic human endogenous retrovirus envelopes identifies syncytin 2, a gene conserved on primate evolution. Proceedings of the National Academy of Sciences. 2003 Oct 28;100(22):13013–8. 10.1073/pnas.2132646100 PMC240736 14557543

[46] Lavillette D, Marin M, Ruggieri A, Mallet F, Cosset FL, Kabat D. The envelope glycoprotein of human endogenous retrovirus type W uses a divergent family of amino acid transporters/cell surface receptors. Journal of Virology. 2002 Jul 1;76(13):6442–52. 12050356 10.1128/JVI.76.13.6442-6452.2002 PMC136247

[47] Blond JL, Lavillette D, Cheynet V, Bouton O, Oriol G, Chapel-Fernandes S, An envelope glycoprotein of the human endogenous retrovirus HERV-W is expressed in the human placenta and fuses cells expressing the type D mammalian retrovirus receptor. Journal of Virology. 2000 Apr 1;74(7):3321–9. 10708449 10.1128/jvi.74.7.3321-3329.2000 PMC111833

[48] Harayama T, Shindou H, Shimizu T. Biosynthesis of phosphatidylcholine by human lysophosphatidylcholine acyltransferase 1. Journal of Lipid Research. 2009 Sep 1;50(9):1824–31. 19383981 10.1194/jlr.M800500-JLR200 PMC2724784

[49] Zou C, Ellis BM, Smith RM, Chen BB, Zhao Y, Mallampalli RK. Acyl-CoA: lysophosphatidylcholine acyltransferase I (Lpcat1) catalyzes histone protein O-palmitoylation to regulate mRNA synthesis. Journal of Biological Chemistry. 2011 Aug 12;286(32):28019–25. 21685381 10.1074/jbc.M111.253385 PMC3151047

[50] Ruvinsky I, Sharon N, Lerer T, Cohen H, Stolovich-Rain M, Nir T, Ribosomal protein S6 phosphorylation is a determinant of cell size and glucose homeostasis. Genes & Development. 2005 Sep 15;19(18):2199–211. 16166381 10.1101/gad.351605 PMC1221890

[51] Delarue M, Brittingham GP, Pfeffer S, Surovtsev IV, Pinglay S, Kennedy KJ, mTORC1 controls phase separation and the biophysical properties of the cytoplasm by tuning crowding. Cell. 2018 Jul 12;174(2):338–49. 29937223 10.1016/j.cell.2018.05.042 PMC10080728

[52] Pollen AA, Bhaduri A, Andrews MG, Nowakowski TJ, Meyerson OS, Mostajo-Radji MA, Establishing cerebral organoids as models of human-specific brain evolution. Cell. 2019 Feb 7;176(4):743–56. 30735633 10.1016/j.cell.2019.01.017 PMC6544371

[53] Plath N, Ohana O, Dammermann B, Errington ML, Schmitz D, Gross C, Arc/Arg3. 1 is essential for the consolidation of synaptic plasticity and memories. Neuron. 2006 Nov 9;52(3):437–44. 17088210 10.1016/j.neuron.2006.08.024

[54] Shepherd JD, Bear MF. New views of Arc, a master regulator of synaptic plasticity. Nature Neuroscience. 2011 Mar;14(3):279. 21278731 10.1038/nn.2708 PMC8040377

[55] Zhang W, Wu J, Ward MD, Yang S, Chuang YA, Xiao M, Structural basis of arc binding to synaptic proteins: implications for cognitive disease. Neuron. 2015 Apr 22;86(2):490–500. 25864631 10.1016/j.neuron.2015.03.030 PMC4409568

[56] Pastuzyn ED, Day CE, Kearns RB, Kyrke-Smith M, Taibi AV, McCormick J, The neuronal gene Arc encodes a repurposed retrotransposon Gag protein that mediates intercellular RNA transfer. Cell. 2018 Jan 11;172(1–2):275–88. 29328916 10.1016/j.cell.2017.12.024 PMC5884693

[57] Pinkstaff JK, Chappell SA, Mauro VP, Edelman GM, Krushel LA. Internal initiation of translation of five dendritically localized neuronal mRNAs. Proceedings of the National Academy of Sciences. 2001 Feb 27;98(5):2770–5. 10.1073/pnas.051623398 PMC30214 11226315

[58] Campillos M, Doerks T, Shah PK, Bork P. Computational characterization of multiple Gag-like human proteins. Trends in Genetics. 2006 Nov 1;22(11):585–9. 16979784 10.1016/j.tig.2006.09.006

[59] Ashley J, Cordy B, Lucia D, Fradkin LG, Budnik V, Thomson T. Retrovirus-like Gag protein Arc1 binds RNA and traffics across synaptic boutons. Cell. 2018 Jan 11;172(1–2):262–74. 29328915 10.1016/j.cell.2017.12.022 PMC5793882

[60] Jönsson ME, Brattås PL, Gustafsson C, Petri R, Yudovich D, Pircs K, Activation of neuronal genes via LINE-1 elements upon global DNA demethylation in human neural progenitors. Nature Communications. 2019 Jul 18;10(1):1–1. 10.1038/s41467-019-11150-8 PMC6639357 31320637

[61] Muotri AR, Marchetto MC, Coufal NG, Oefner R, Yeo G, Nakashima K, L1 retrotransposition in neurons is modulated by MeCP2. Nature. 2010 Nov;468(7322):443–6. 21085180 10.1038/nature09544 PMC3059197

[62] Liu L, Leng L, Liu C, Lu C, Yuan Y, Wu L, An integrated chromatin accessibility and transcriptome landscape of human pre-implantation embryos. Nature Communications. 2019 Jan 21;10(1):1–1. 10.1038/s41467-018-08244-0 PMC6341076 30664750

[63] Kuwabara T, Hsieh J, Muotri A, Yeo G, Warashina M, Lie DC, Wnt-mediated activation of NeuroD1 and retro-elements during adult neurogenesis. Nature Neuroscience. 2009 Sep;12(9):1097–105. 19701198 10.1038/nn.2360 PMC2764260

[64] Orqueda AJ, Gatti CR, Ogara MF, Falzone TL. SOX-11 regulates LINE-1 retrotransposon activity during neuronal differentiation. FEBS Letters. 2018 Nov;592(22):3708–19. 30276805 10.1002/1873-3468.13260

